# The International Medical Annual and Practitioner’s Index

**Published:** 1899-06

**Authors:** 


THE INTERNATIONAL MEDICAL ANNUAL AND PRACTITIONER'S INDEX. A work of reference for modern practitioners. Seventeenth year. New York: E. B. Treat, 241–243 W. 23d street. 1899.

The annual for 1899 is superior in excellence to any of its predecessors; the volume is larger (760 pages), better illustrated (15 full-page colored plates, and 12 full-page half-tone plates, besides numerous illustrations in the text.) The text has been exceptionally well edited and contains several original articles of special importance by experts in their special departments.

Among the special articles will be found the following: Practical x-ray work, by Norris Wolfenden; Advances in skull surgery, by Seneca D. Powell; Surgical treatment of paralysis, by Robert Jones and A. H. Tubby. These articles are freely illustrated, chiefly by reproductions and photographs. An excellent article on Climatic treatment of consumption, by F. de Havilland Hall, as well as one on Legal decisions affecting medical men, by William A. Purrington, will be found interesting and pertinent. In response to the request of many subscribers there will be found an article on The chief pathogenic bacteria in the human subject, with descriptions of their morphology and methods of microscopical examination, by S. G. Shattock, the pathological curator of the museum of the Royal College of Surgeons, London, illustrated by a series of finely colored plates.

The department of therapeutics, new treatment and sanitation are abreast of the times, and we feel certain that no medical work of such a widely international character has been previously issued by the medical press in any country which offers so much at so small a price. In typography the volume is similar to Treat's well-known medical classics. W. C. K.

